# Estimating the burden of influenza‐related and associated hospitalizations and deaths in France: An eight‐season data study, 2010–2018

**DOI:** 10.1111/irv.12962

**Published:** 2022-01-10

**Authors:** Magali Lemaitre, Fayssoil Fouad, Fabrice Carrat, Pascal Crépey, Jacques Gaillat, Gaëtan Gavazzi, Odile Launay, Anne Mosnier, Marie‐Cécile Levant, Mathieu Uhart

**Affiliations:** ^1^ IQVIA Courbevoie France; ^2^ Sorbonne Université, Institut National de la Santé et de la Recherche Médicale (Inserm), Institut Pierre Louis d'Epidémiologie et de Santé Publique Paris France; ^3^ Assistance Publique‐Hôpitaux de Paris (AP‐HP), Hôpital Saint‐Antoine, Unité de Santé Publique Paris France; ^4^ Université de Rennes, EHESP, REPERES—EA 7449 Rennes France; ^5^ Centre Hospitalier Annecy‐Genevois Metz‐Tessy France; ^6^ CHU Grenoble Alpes, Clinique Universitaire de Gériatrie, Pavillon Elisée‐Chatin, and GREPI EA 7408, Université Grenoble Alpes Grenoble France; ^7^ Faculté de Médecine Paris Descartes Université de Paris Paris France; ^8^ Inserm, CIC 1417, F‐CRIN I‐REIVAC, Assistance Publique‐Hôpitaux de Paris, CIC Cochin‐Pasteur Paris France; ^9^ Open Rome & Réseau des GROG Paris France; ^10^ Sanofi Pasteur Lyon France

**Keywords:** cost of illness, epidemiology, France, hospitalization, human, mortality

## Abstract

**Background:**

In France, each year, influenza viruses are responsible for seasonal epidemics leading to 2–6 million cases. Influenza can cause severe disease that may lead to hospitalization or death. As severe disease may be due to the virus itself or to disease complications, estimating the burden of severe influenza is complex. The present study aimed at estimating the epidemiological and economic burden of severe influenza in France during eight consecutive influenza seasons (2010–2018).

**Methods:**

Influenza‐related hospitalization and mortality data and patient characteristics were taken from the French hospital information database, PMSI. An ecological approach using cyclic regression models integrating the incidence of influenza syndrome from the Sentinelles network supplemented the PMSI data analysis in estimating excess hospitalization and mortality (CépiDc—2010–2015) and medical costs.

**Results:**

Each season, the average number of influenza‐related hospitalizations was 18,979 (range: 8627–44,024), with an average length of stay of 8 days. The average number of respiratory hospitalizations indirectly related with influenza (i.e., influenza associated) was 31,490 (95% confidence interval [CI]: 24,542–39,012), with an average cost of €141 million (range: 54–217); 70% of these hospitalizations and 77% of their costs concerned individuals ≥65 years of age (65+). More than 90% of excess mortality was in 65+ subjects.

**Conclusions:**

The combination of two complementary approaches allowed estimation of both influenza‐related and associated hospitalizations and deaths and their burden in France, showing the substantial impact of complications. The present study highlighted the major public health burden of influenza and its severe complications, especially in 65+ subjects.

## 1 INTRODUCTION

In high‐income countries, influenza is one of the most important illnesses affecting individuals of all ages. Seasonal epidemics are currently due to type A, subtype A(H1N1) or A(H3N2), or type B (B/Victoria and B/Yamagata lineages). Studies showed that A(H3N2) viruses usually affect the elderly (≥65 years: 65+) more frequently, whereas A(H1N1) viruses usually affect younger subjects.^1^, ^2^ Every year, influenza leads to 3 to 5 million severe cases and the death of 240,000–650,000 persons.^3^ In France, each year, 2 to 6 million people are affected by influenza, leading on average to 2.5 million medical consultations and 10,000 excess deaths, 90% of which occurring in 65+.^4^

Most infected subjects have a mild form of the disease and do not require medical attention, but serious complications may occur, mainly in populations with a higher risk of serious illness due to aging or underlying chronic conditions. Complications result from the influenza virus infection itself, or from viral or bacterial superinfection (pneumonia) or organic decompensation, mainly in patients with neurologic and cardiovascular comorbidities.^1^, ^5^, ^6^ Consequently, influenza is not systematically reported on the patient's chart or death certificate, leading to underestimation of the global burden of influenza. Some ecological studies assessed the question of the indirect burden (i.e., excess burden approach) of influenza, which was commonly estimated using mortality data, but less frequently using morbidity data.^7^, ^8^

According to the World Health Organization, vaccination is the most effective way to prevent infection and severe outcomes caused by influenza viruses.^9^ Several immunization programs are implemented in Europe, the most common being the French program to protect healthcare workers and at‐risk individuals: the elderly (65+), individuals ≥6 months but with chronic conditions, people with morbid obesity, and pregnant women. Measures to decrease transmission are also implemented during epidemics, such as handwashing or masks. Estimating the global influenza burden in terms of excess hospitalization and death and the economic consequences is therefore important for decision‐makers, to optimize prevention policies. A recent study in the United States estimated that the average annual total economic burden of influenza was as high as $11.2 billion.^6^

In the present study, the epidemiological and economic burden of hospitalization and in‐hospital mortality directly due to influenza (i.e., influenza related) as calculated from the French healthcare databases^7^, ^10^ was supplemented by the burden calculated from excess hospitalization and mortality indirectly due to influenza (influenza associated) and related costs.

## 2 METHODS

To evaluate burden of disease, we used two approaches. The first was the evaluation of the burden of influenza in the hospital, in terms of the number of influenza‐related hospitalizations and in‐hospital deaths. The second was the evaluation of the burden of influenza in the hospital, in terms of influenza‐associated excess hospitalization and mortality.

### 2.1 Data and data sources

The study was conducted using the French hospital databases named PMSI recording medical information about all the hospitalizations performed annually and covering reimbursement information for each public or private hospital since 2007. PMSI is part of the SNDS national health data system database, and the exhaustive CépiDc (Epidemiology Center of Medical Causes of Death) database of all French deaths. The French regulation allows the use of PMSI database with a historic of 10 years; consequently, data from the PMSI were explored for the period 2010–2018. Due to the time period to collect and clean death certificate, data from the CépiDc were available for the period 2010–2015.

*For hospitalization data (morbidity)*, exhaustive data from private and public hospitals, with a single patient identifier, were collected in the PMSI Discharge Database. The two subdatabases required in the analysis collected information from facilities practicing Medicine, Surgery and Obstetrics (PMSI‐MCO), and from rehabilitation and recuperative care facilities (PMSI‐SSR). For the direct approach, we selected patients with at least one hospital stay in PMSI‐MCO in France during the 2010–2018 period with a principal diagnosis (PD), a related diagnosis (RD), or an associated diagnosis (AD) coded as J09 (influenza due to certain identified influenza viruses), J10 (influenza due to other identified influenza virus), or J11 (influenza due to unidentified influenza virus) according to the International Classification of Diseases 10th Revision (ICD‐10).^10^ For the excess burden approach, weekly data of hospital stays between 2010 and 2018 were extracted from the PMSI‐MCO, and the cost corresponding to each hospital stay was derived. Hospitalization data (PD) from pneumonia and influenza (P&I) (ICD‐10 codes: J09–J18), respiratory diseases (ICD‐10 codes: J00–J99), cardiovascular diseases (ICD‐10 codes: CIM‐10 I00–I99), and all causes based on the ICD‐10 were compiled.

*For mortality data*, in the direct approach, all deaths occurring during a hospital stay for influenza (ICD‐10 codes: J09–J11) were included. In the excess burden approach, weekly age‐specific underlying cause‐of‐death data for the whole French population were obtained from death certificates collected by Inserm‐CépiDc from June 2010 through June 2015 (last year with available data).^11^ We compiled deaths from P&I (ICD‐10 codes: J09–J18), respiratory diseases (ICD‐10 codes: J00–J99), cardiovascular diseases (ICD‐10 codes: CIM‐10 I00–I99), and all causes based on the ICD‐10.

*For demographic data*, we used yearly population data obtained from the National Institute of Statistics and Economic Studies (INSEE) to calculate age‐standardized hospitalization or death rates,^12^ using the June 2017 French population structure as a reference.

*Influenza‐like‐illness (ILI)* incidence in conjunction with documented cocirculation of influenza viruses has been shown to be a good proxy for influenza incidence in France and elsewhere^13^ and an appropriate covariate in mortality burden models.^14^ Weekly ILI incidence was obtained from the French Sentinelles surveillance network from 2011 to 2018. This surveillance network relies on volunteer general practitioners (1% of all general practitioners), who have been reporting medical consultations for ILI and other infections since 1984.^5^ The ILI case definition consisted of a combination of fever >39°C, myalgia, and respiratory symptoms as per the French Sentinelles definition.^15^

### 2.2 Statistical approach

For each approach, analysis of hospitalizations was conducted for 2010–2018 and analysis of mortality for 2010–2015; both analyses were stratified by age group in two ways: (a) 0–4, 5–19, 20–49, 50–64, and ≥65 years or (b) <65 years (65−) and ≥65 years (65+).

**For the epidemiological burden**, *in the direct burden approach*, the incidence and the number and proportion of patients hospitalized for influenza (DP‐DR coded with ICD‐10 J09–J11 or DAS coded with ICD‐10 J09–J11)^16^ were estimated. To estimate the mortality burden of influenza, in‐hospital deaths for all causes were counted for each July–June respiratory season and expressed as a hospital mortality rate. *In the excess burden approach*, several indirect approaches have traditionally been used to estimate excess mortality due either to influenza or to its complications, but the preferred model was to explicitly model weekly mortality data against weekly indicators of influenza activity.^15^ This ecological approach,^15^ commonly used to estimate mortality impact, was applied here to both mortality and hospitalization data.^17^, ^18^, ^19^ Precisely, different Poisson cyclic models (time series) were built, where age‐ and cause‐specific hospitalization as well as mortality data were explained by ILI incidence,^17^, ^19^, ^20^, ^21^ time trends, and seasonal terms, using a log link.^8^, ^14^ A stepwise selection method was used to identify the significant time trends and seasonal terms in the age‐ and cause‐specific models. The model was fitted to data from 2000 to 2015 for mortality and from 2010 to 2018 for hospitalizations. Baseline hospitalizations and mortality were calculated from the Poisson models as the expected values when the ILI variable was set to zero. The weekly number of excess hospitalizations and excess mortality due to influenza was estimated as the difference between the expected number and mortality from the full model and baseline hospitalizations and mortality, respectively. Then, weekly excess hospitalization and mortality were summed for each July–June respiratory season to obtain excess hospitalization and mortality estimates.

**For the economic burden**, *the direct burden approach* was based on the resources used during direct influenza hospitalizations coded in the PMSI. Total and average costs per stay were calculated by year from public health expenditure perspective, considering the French national health insurance reimbursement, public and private complementary insurance, and out‐of‐pocket charges. *In the excess burden approach*, the influenza‐associated burden was calculated on the basis of influenza‐associated excess hospitalization and the average cost per hospital stay for the different causes of hospitalization. Prior to estimation of economic burden, we estimated the average cost per hospital stay for each of these causes: P&I, respiratory causes, cardiovascular causes, and all causes. The average cost was based on use of all economic resources associated with a hospitalization, for a given cause, recorded in the PMSI during the winter months (December to March). To calculate the economic burden indirectly attributable to influenza for a given cause, we applied the average cost per hospitalization to influenza‐associated excess hospitalizations.

## 3 RESULTS

### 3.1 Epidemiological burden of influenza

#### 3.1.1 Direct burden approach

Between seasons 2010–2011 and 2017–2018, the number of influenza‐related hospitalizations ranged from 8627 to 44,024, for an average 18,979, corresponding to incidence of 28 per 100,000. The eight epidemic seasons were distributed as follows: (a) three seasons of low intensity (<10,000 influenza‐related hospitalizations), that is, 2010–2011 due to A(H1N1)/B, 2011–2012 due to A(H3N2), and 2013–2014 due to A(H1N1)/(H3N2); (b) two seasons of middle intensity (10,000–20,000), that is, 2012–2013 due to A(H1N1)/A(H3N2)/B and 2015–2016 due to A(H1N1)/B; and (c) three seasons of severe intensity (>20,000), that is, 2014–2015 due to A(H1N1)/A(H3N2)/B, 2016–2017 due to A(H3N2), and 2017–2018 due to A(H1N1)/B (Table 1). Incidence varied between 13 and 63 per 100,000 persons.

**TABLE 1:** Number of influenza‐related hospitalizations by age group and epidemic season in France, 2010–2018 seasons

Epidemic season	Strain	Age group (years), N (%)							All ages
		0–4	5–19	20–49	50–64	65–74	75–84	≥85	
2010–2011	H1/B	3160 (31.8)	1580 (15.9)	2579 (26)	1251 (12.6)	525 (5.3)	507 (5.1)	332 (3.3)	9934 (100.0)
2011–2012	H3	2561 (29.2)	811 (9.3)	1548 (17.7)	856 (9.8)	710 (8.1)	1190 (13.6)	1077 (12.3)	8753 (100.0)
2012–2013	H3 mixed	3641 (27.3)	1637 (12.2)	2791 (20.9)	1880 (14.1)	1069 (8.0)	1306 (9.8)	1035 (7.7)	13,359 (100.0)
2013–2014	H3 mixed	2267 (26.3)	715 (8.3)	2092 (24.2)	1365 (15.8)	784 (9.1)	860 (10.0)	544 (6.3)	8627 (100.0)
2014–2015	H3 mixed	4108 (19.4)	1411 (6.7)	3123 (14.8)	2825 (13.4)	2346 (11.1)	3717 (17.6)	3602 (17.0)	21,132 (100.0)
2015–2016	H1/B	5314 (29.5)	2313 (12.9)	3362 (18.7)	2201 (12.2)	1911 (10.6)	1770 (9.8)	1137 (6.3)	18,008 (100.0)
2016–2017	H3	3107 (11.1)	1230 (4.4)	2451 (8.8)	2697 (9.6)	3619 (12.9)	6556 (23.4)	8335 (29.8)	27,995 (100.0)
2017–2018	H1/B	7749 (17.6)	2414 (5.5)	4891 (11.1)	5995 (13.6)	6115 (13.9)	8332 (18.9)	8528 (19.4)	44,024 (100.0)
All epidemic seasons		31,907 (21.0)	12,111 (8.0)	22,837 (15.0)	19,070 (12.6)	17,079 (11.2)	24,238 (16.0)	24,590 (16.2)	151,832 (100.0)

During the whole study period, the youngest (0–4 years) and oldest (65+: i.e., 65–74, 75–84, and ≥85 years) age groups accounted a majority of influenza‐related hospitalizations (64%, of which 21% in the 0–4 group). Variability was high from one season to another; in 2016–2017, which was of severe intensity, the 75+ group accounted for 53% of hospitalizations (Table 1).

During the whole study period, median hospital stay was threefold higher for 85+ (median: 10 days; range: [9–10]) than 0–4 (3 days; [3–3]) (Table 2). The proportion of intensive care unit transfer ranged from 0.0% [0.0–0.1] for the 0–4 age group to 23.1% [18.2–26.9] for the 50–64 age group. The elderly population (i.e., 65+) accounted for 80% of in‐hospital deaths from influenza (Table 2).

**TABLE 2:** Summary of the characteristics of the hospitalizations for influenza per age group in France, 2010–2018 seasons

	Age group (years)							All ages
	0–4	5–19	20–49	50–64	65–74	75–84	≥85	
Median length of hospital stays (days)								
Median	3	3	4	7	8	8	10	5
[Range]	[3–3]	[3–3]	[3–4]	[6–7]	[7–8]	[8–9]	[9–10]	[4–7]
Emergency unit transfer proportion^a^ (%)								
Median	18.6	22.7	31.2	22.0	21.8	23.7	25.2	23.6
[Range]	[17.1–20.2]	[21.9–31.0]	[30.1–33.0]	[18.7–25.4]	[20.0–23.2]	[21.3–24.9]	[22.8–25.6]	[22.3–24.6]
Intensive care unit transfer proportion^a^ (%)								
Median	0.0	1.2	13.2	23.1	19.8	13.8	6.3	11.4
[Range]	[0.0–0.1]	[1.2–1.7]	[10.0–15.8]	[18.2–26.9]	[17.6–23.4]	[11.1–16.9]	[5.3–6.9]	[9.5–12.8]
In‐hospital death proportion (%)								
Median	0.1	0.4	1.0	4.1	6.1	6.6	9.3	2.9
[Range]	[0.0–0.2]	[0.1–0.7]	[0.4–1.8]	[2.3–5.5]	[3.9–6.9]	[5.1–7.5]	[7.1–11.6]	[2.1–5.2]
3‐month readmission proportion (%)								
All causes								
Median	13.4	13.1	23.9	25.8	27.7	26.0	21.9	20.6
[Range]	[11.5–14.5]	[11.6–28.3]	[23.4–28.6]	[23.2–28.9]	[25.4–28.9]	[24.4–28.0]	[20.6–24.2]	[19.5–23.7]
Respiratory cause								
Median	5.0	2.3	2.8	6.6	6.8	6.0	5.5	4.8
[Range]	[4.3–5.3]	[1.4–3.0]	[2.1–7.1]	[5.7–7.5]	[5.9–7.5]	[5.1–7.4]	[4.3–6.0]	[4.4–5.5]
Cardiac cause								
Median	0.1	0.3	1.2	4.6	6.3	6.3	5.8	2.8
[Range]	[0.1–0.7]	[0.2–2.2]	[1.1–6.0]	[4.4–6.9]	[5.1–8.8]	[5.6–8.8]	[5.2–6.4]	[2.0–5.1]

^a^ Data available only from the epidemic season 2012–2013.

Among patients hospitalized for influenza who did not die during the stay, all ages combined and over the entire period, the median proportion of rehospitalization for all causes within 3 months was 20.6% [19.5–23.7] (Table 2). This proportion ranged from 13.1% [11.6–28.3] for the 5–19 age group to 27.7% [25.4–28.9] for the 65–74 age group. Rehospitalization was often related to respiratory causes (median proportion: 4.8%), followed by cardiac causes (median proportion: 2.8%) (Table 2).

#### 3.1.2 Excess burden approach or burden of influenza‐associated hospitalization and death

*Regarding performance of the model and model fitting*, the hospitalization models showed good performance, with high correlations between observed and predicted values and low mean absolute percentage error (MAPE) for P&I hospital stays and respiratory causes regardless of age group (Table S1 and Figure S1). The fit between the observed and predicted values for cardiac and all causes (Figure S1) was lower. This was particularly true among younger subjects.

The mortality models showed high correlation between observed and predicted values and MAPE for the older age groups for P&I, respiratory, cardiac, and all causes. For younger subjects (0–4 and 5–19 age groups), correlations and MAPE were weaker (correlations between 0.48 and 0.76 and MAPE between 44% and 54%), compared with older age groups (Table S1).

*Regarding estimated excess influenza‐associated hospitalization*, the standardized P&I and respiratory excess hospitalization rates were always higher in 65+ compared with all ages taken together.

The proportions of excess influenza‐associated hospitalization during winter months (December to March) estimated for all ages and during the severe epidemic seasons were on average 33% for P&I and 14% for respiratory causes. For the other seasons, proportions were on average 18% and 8.1%, respectively. In 65+, during the severe epidemic seasons (2014–2015, 2016–2017, and 2017–2018), the proportions were on average 34% and 23% for P&I and respiratory causes, respectively (Table 3).

**TABLE 3:** Estimated excess of influenza‐associated hospitalization per epidemic season in France, 2010–2018 seasons

Influenza seasons	All ages						65+					
	P&I			Respiratory causes			P&I			Respiratory causes		
	N	95% CI	Rate^a^	N	95% CI	Rate^a^	N	95% CI	Rate^a^	N	95% CI	Rate^a^
2010–2011	8140	(6019–10,332)	12.9	17,728	(10,543–25,199)	28.1	1082	(−122 to 2366)	10.1	2809	(748–5039)	26.3
2011–2012	17,896	(16,046–19,825)	28.2	33,258	(26,392–40,189)	52.5	13,219	(12,221–14,304)	120.5	24,713	(22,740–26,775)	225.2
2012–2013	18,475	(16,415–20,658)	29.0	24,117	(17,106–31,704)	37.9	11,164	(9975–12,488)	98.8	20,868	(18,632–23,226)	184.6
2013–2014	7402	(5334–9505)	11.6	13,071	(6564–20,191)	20.4	3469	(2265–4701)	29.8	6865	(4576–9146)	58.9
2014–2015	27,592	(25,900–29,313)	43.1	39,257	(33,271–46,194)	61.3	19,160	(18,173–20,187)	159.8	32,162	(30,243–34,283)	268.3
2015–2016	20,691	(18,499–23,137)	32.1	29,012	(21,396–36,672)	45.0	8742	(7410–10,280)	71.0	14,925	(12,494–17,667)	121.2
2016–2017	30,939	(28,978–32,996)	47.9	48,672	(42,110–55,543)	75.3	25,511	(24,354–26,803)	202.6	38,833	(36,724–41,161)	308.3
2017–2018	40,045	(37,260–42,688)	61.9	46,809	(38,957–56,407)	72.3	25,627	(23,954–27,240)	199.0	35,516	(32,373–38,721)	275.8

Abbreviations: CI, confidence interval; N, number of events; P&I, pneumonia and influenza.

^a^ Rate per 100,000 persons.

**FIGURE 1:**
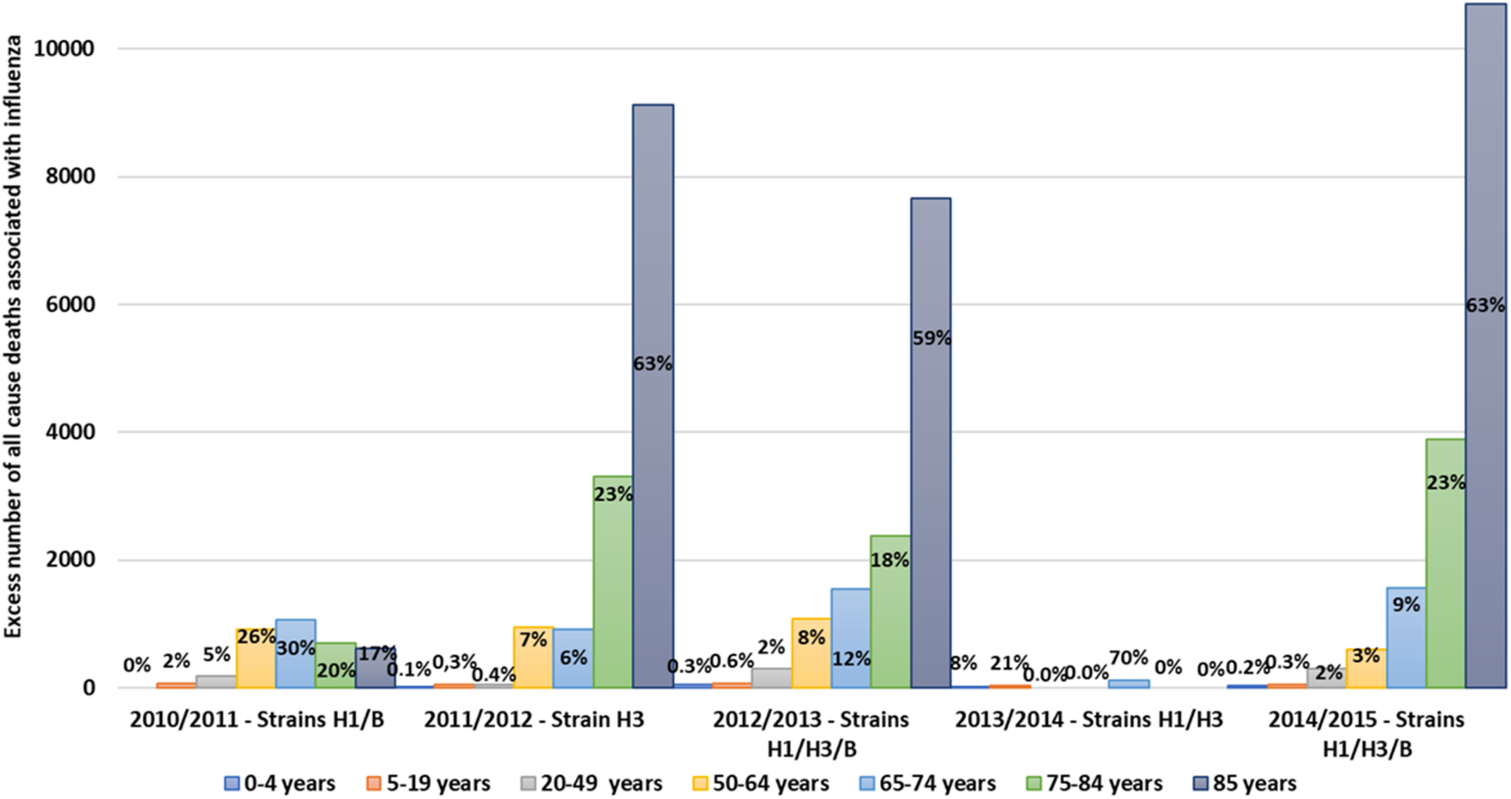
Estimated excess of influenza‐associated mortality per age group and epidemic season in France, 2010–2015 seasons

*Regarding estimated excess of influenza‐associated mortality*, in the 65+, taking all seasons together, the estimated average excess *of influenza‐associated mortality* was 8709 deaths (95% confidence interval [CI]: 6722–11,152); it was 16,139 (95% CI: 13,472–18,867) for the 2014–2015 season, which was of severe intensity. The population of 65 years and more accounted for an average of 83% and 95% of the whole *influenza‐associated mortality* for the 2010–2015 period and the 2014–2015 season, respectively (Figure 1). The corresponding rates were 78 and 135 per 100,000 respectively. In the elderly, the contribution of influenza to mortality during winter months (December to March), estimated on average for all the epidemic seasons and during the severe 2014–2015 season, was 19% and 35% in P&I, respectively, 14% and 25% in respiratory causes respectively, and 5.6% and 10% in all causes, respectively. During this severe epidemic season, the excess death rates attributable to influenza were of 20 (P&I), 35 (respiratory causes), and 125 (all causes) per 100,000 (Table S2).

### 3.2 Economic burden of influenza

#### 3.2.1 Direct burden approach

**FIGURE 2:**
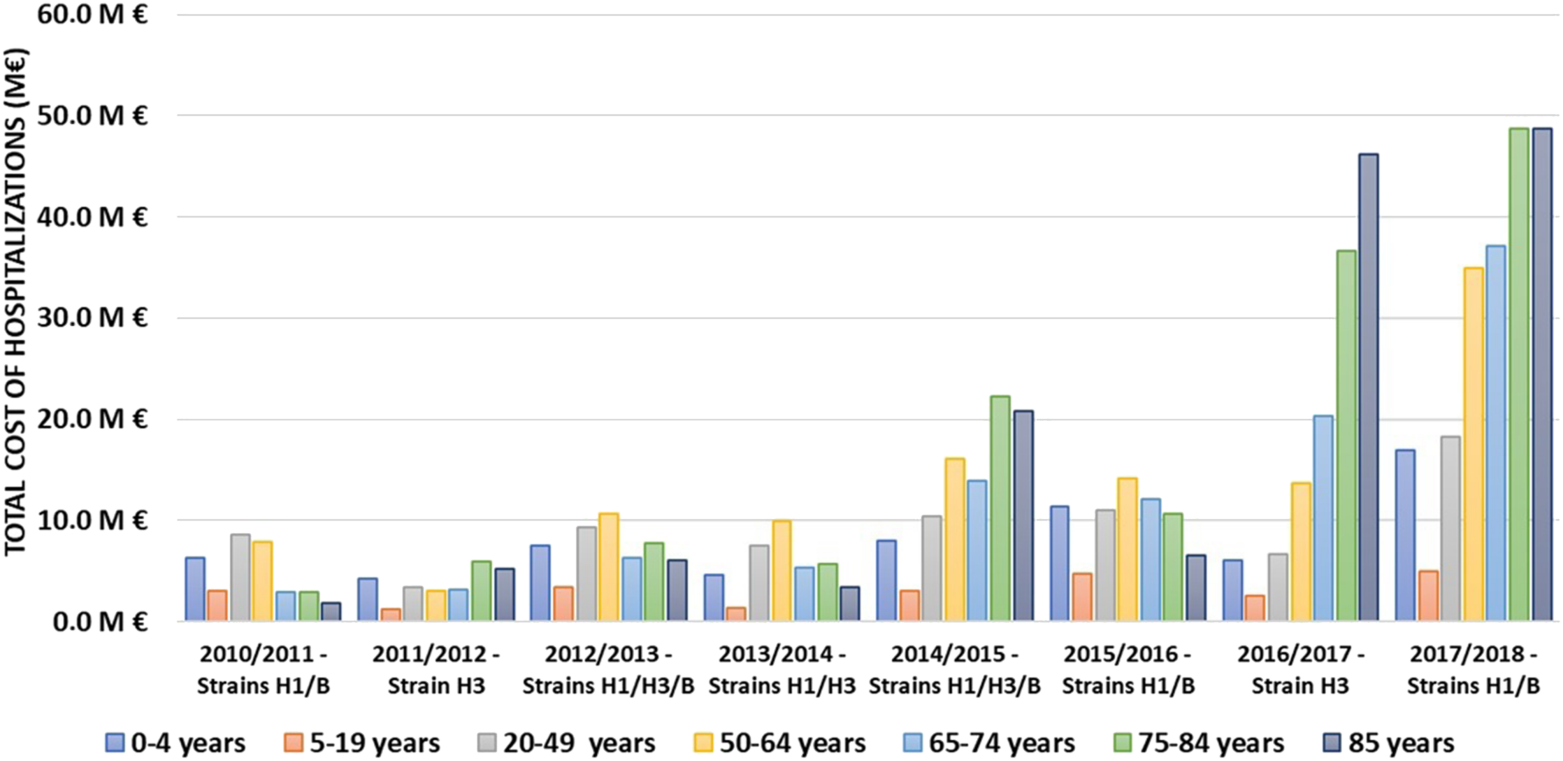
Total direct cost of all influenza‐related hospital stays per epidemic season and age group in France, 2010–2018 seasons

Costs varied substantially depending on the epidemic season, from €26.4 million in 2011–2012 to €209.8 million in 2017–2018. During low‐ and middle‐intensity epidemic seasons, total cost for all ages averaged €43.9 million, with relatively balanced distribution between age groups. The highest total cost during these seasons was in the 50–64 age group, with an average €9.1 million, and the lowest in the 5–19 age group, with €2.8 million. The severe epidemic seasons showed a different pattern, with an average total cost for all ages of €145.5 million; 65+ accounted for more than 67% of the total cost, with an average of €98.3 million (Figure 2).

#### 3.2.2 Indirect approach or burden of influenza‐associated hospitalization and death

The P&I and respiratory excess costs attributable to influenza varied from €26.8 and €54.1 million respectively in 2010–2011 to €173.1 and €217.4 million in 2017–2018 (Table 4). During the severe epidemic seasons, total cost was 4‐fold to 5.5‐fold higher in 65+ than 65− (€171.8 million vs. €35.0 million for respiratory causes) (Table 4).

**TABLE 4:** Total cost of excess hospitalizations attributed to influenza for P&I and respiratory causes, by age group and influenza season in France, 2010–2018 seasons

	Influenza seasons															
	2010–2011		2011–2012		2012–2013		2013–2014		2014–2015		2015–2016		2016–2017		2017–2018	
	Age group															
	65−	65+	65−	65+	65−	65+	65−	65+	65−	65+	65−	65+	65−	65+	65−	65+
P&I (M€)	21.5	5.3	13.3	65.2	25.2	14.9	17.3	17.3	28.4	92.1	39.2	43.2	17.6	121.8	48.8	124.3
Respiratory causes (M€)	40.6	13.5	22.4	120.9	25.6	105.2	23.6	34.8	31.5	155.5	48.5	74.8	29.6	186.4	43.8	173.6

Abbreviation: P&I, pneumonia and influenza.

In 65+, for the severe epidemic seasons, total excess costs attributable to influenza accounted for 34% and 23% of the elderly P&I and respiratory total costs, respectively.

## 4 DISCUSSION

To the best of our knowledge, this was the first national study to integrate two complementary direct and indirect approaches to estimate the global hospital epidemiological and economical morbidity and mortality burden of influenza. The first approach was conducted directly from national hospitalization data and the second approach from modeling of the excess hospitalization and mortality associated with influenza.

Temporal variability was observed over the study period (2010–2018), with different intensity levels in the eight epidemic seasons; the average hospitalization rate during the three seasons of severe intensity was around twofold higher than during the five seasons of low or moderate intensity. The rate during severe seasons was particularly high for the 65+, with 126 hospitalizations per 100,000 on average, compared with 26 per 100,000 in the 65−. This variation over seasons and the greater vulnerability of the elderly population are well‐known worldwide.^22^, ^23^, ^24^, ^25^

Like in other studies, influenza epidemics due to H1N1 or H1N1/B affected particularly young people, whereas H3N2 strains affected particularly elderly people, where they were associated with high mortality.^26^ In addition, neither median hospital stays nor the proportion of mortality directly related to the influenza hospitalization varied with epidemic severity. However, influenza can lead to complications in vulnerable people, including the elderly, sometimes some weeks after the actual influenza episode, and in this case, the death is not officially associated with influenza in the death certificate.^27^ This was the reason for our indirect analysis (cyclic regression), commonly used to estimate the impact of influenza on mortality in the elderly.^17^, ^20^, ^28^, ^29^, ^30^, ^31^ This provided substantial complementary information and refined the results of the direct approach, by showing a consistent relationship between the excess mortality attributable to influenza and the severity of the epidemic. Less than 20% of deaths were identified by the direct approach in the 65+, whereas they accounted for 90% of the estimated all‐cause mortality attributable to influenza, showing the importance of the indirect burden in this vulnerable population. Results confirmed findings from other studies in high‐ and middle‐income countries, which suggested a moderate contribution of influenza to total winter mortality among seniors, estimated at around 5% (range 2.2–16%) in the present study.^25^, ^32^, ^33^ Finally, findings suggested a substantial contribution to respiratory winter mortality (around 14%).

We applied those models to estimate the excess hospitalization associated with influenza. The most specific cause of influenza‐associated hospitalization (P&I) showed a very good fit between the direct (hospital stays directly attributed to influenza) and indirect approach (rate of 12 vs. 13 and 62 vs. 63 per 100,000 for 2013–2014 and 2017–2018, respectively). This is a strong argument in favor of our model's validity, at least for P&I and respiratory causes. On the other hand, the lower precision of the estimates of the cardiac and all‐cause models suggests that these models were not specific enough to provide robust estimates of the excess influenza‐associated hospitalization for cardiac causes and all causes. Such models have been found to be relevant to predict the cardiac hospitalization excess, but mostly in 80+.^28^

Overall, the direct burden corresponded to 55% excess hospitalization attributable to influenza for all ages, but only 33% for the 65+.

The study further assessed the economic counterpart of both the epidemiological burden and excess hospitalization directly and indirectly associated with influenza. Consistently, associated costs differed with epidemic severity, with a cost 3–4 times higher on average in severe than low or moderate severity seasons (all ages). In agreement with the epidemiological burden, this trend was even more marked in the elderly population, which accounted for 83% of costs during severe epidemic seasons. In addition, the direct cost of hospitalization represented 34% of the cost estimated by the indirect approach for the low or moderate severity epidemics. However, during severe epidemics, this proportion was on average 56% in the 65+, showing consistently that most hospitalizations were attributed to influenza during the severe epidemics.

Our study had some limitations. The excess hospitalization and mortality model used ILI incidence as an indicator of influenza activity, which may be affected by greater vaccine coverage in seniors. The proportion of positive influenza virus tests would have also been a valid indicator of influenza activity, but these data were not available for the entire study period. In addition, there may be some overlap with other virus epidemics such as respiratory syncytial virus (RSV) (depending on the year) and could be integrated in the model. Nonetheless, comparison of models using ILI and percentage positive influenza tests demonstrated that ILI was the most statistically relevant indicator for mortality and, most importantly, that both indicators produce similar influenza burden estimates in France.^14^

In conclusion, our study provided a comprehensive description of the burden of severe influenza in France. It provided an estimate of the proportion hospitalizations indirectly related (i.e., associated) with influenza, highlighting the importance of considering this indirect approach when investigating the burden of influenza. The study further highlighted the complexity of estimating the global burden of influenza, whether epidemiological or economic. Influenza virus virulence is difficult to predict, and it is difficult to estimate the impact of influenza‐associated complications (e.g., cardiovascular events) in the elderly (85+).^34^ Further studies should assess the organizational impact of influenza epidemics (overwork in hospital departments, increased sick leave in the general population, …), which is an important aspect that was not evaluated in the present study.

## AUTHOR CONTRIBUTIONS

**Magali Lemaitre:** Conceptualization; methodology. **Fayssoil Fouad:** Formal analysis. **Fabrice Carrat:** Methodology; validation. **Pascal Crépey:** Methodology; validation. **Jacques Gaillat:** Methodology; validation. **Gaëtan Gavazzi:** Methodology; validation. **Odile Launay:** Methodology; validation. **Anne Mosnier:** Methodology; validation. **Mathieu Uhart**: Conceptualization; methodology; validation. **Marie‐Cécile Levant:** Conceptualization; methodology; validation.

### PEER REVIEW

The peer review history for this article is available at https://publons.com/publon/10.1111/irv.12962.

## Supporting information


**Appendix S1:** Supporting Information

**Table S1:** Performance of the models used to estimate excess influenza‐associated hospitalization and mortality
**Table S2:** Estimated excess influenza‐related mortality per cause and age group in France, 2010–1015 seasons

**Figure S1:** Weekly rates for P&I, respiratory, cardiac, and all‐causes hospitalizations per age groups in France, July 2010–June 2018.
Observed hospitalization rates (dashed blue line) and predicted hospitalization rates (blue line) based on a Poisson model integrating seasonal terms, time trends, and influenza‐like‐illness rates. Baseline morbidity rates predicted by the Poisson model in the absence of influenza activity are indicated by a dark blue area.

## Data Availability

The data that support the findings of this study are available from the corresponding author upon reasonable request.
