# Supplementary material for: Estimating the burden of influenza‐related and associated hospitalizations and deaths in France: An eight‐season data study, 2010–2018
Source: Influenza Other Respir Viruses. 2022 Jan 10;16(4):717–25. doi: 10.1111/irv.12962 (PMC9178052; doi:10.1111/irv.12962)
Supplement: Supplementary file 1 — Appendix S1: Supporting Information [file IRV-16-717-s002.docx]

Appendices

## Supporting Information

To estimate excess burden attributable to influenza, we fit Poisson models with a log link to weekly hospitalization or mortality data during 2010-2018 and 2010-2015 respectively.

Different Poisson cyclic models (time series) were built, where age- and cause-specific hospitalization and mortality data were explained by ILI incidence [17,19–21], time trends and seasonal terms, using a log link [10,14] as follows :

Full model:

Log(E(Yt/Pop)) = β0 + β1 Xflu(t) * Year_i + β2*t + β3*t2 + β4 t3 + β5*sin(2*Pi*t/52.17) + β6*cos(2*Pi*t/52.17) + β7*sin(3*Pi*t/52.17) + β8*cos(3*Pi*t/52.17) + β9*sin(4*Pi*t/52.17) + β10*cos(4*Pi*t/52.17) ) + β11*sin(6*Pi*t/52.17) + β12*cos(6*Pi*t/52.17) + β13*sin(8*Pi*t/52.17) + β14*cos(8*Pi*t/52.17) + β15*sin(10*Pi*t/52.17) + β16*cos(10*Pi*t/52.17) + β17*sin(12*Pi*t/52.17) + β18*cos(12*Pi*t/52.17)

Where Yt represents weekly number of respiratory deaths for a specific age group and cause of hospitalization / death at week t, Pop is a population offset, t. t2 and t3 are linear, quadratic and cubic terms for time trend, sin and cos are harmonic terms representing seasonality, Xflu represents the moving average of ILI incidence, and Year_i is a dummy variable representing each of the respiratory year studied, i=1…13.

Baseline(t)= β0 + β2*t + β3*t2 + β4 t3 + β5*sin(2*Pi*t/52.17) + β6*cos(2*Pi*t/52.17) + β7*sin(3*Pi*t/52.17) + β8*cos(3*Pi*t/52.17) + β9*sin(4*Pi*t/52.17) + β10*cos(4*Pi*t/52.17) ) + β11*sin(6*Pi*t/52.17) + β12*cos(6*Pi*t/52.17) + β13*sin(8*Pi*t/52.17) + β14*cos(8*Pi*t/52.17) + β15*sin(10*Pi*t/52.17) + β16*cos(10*Pi*t/52.17) + β17*sin(12*Pi*t/52.17) + β18*cos(12*Pi*t/52.17)

The baseline model comprised significant trend and seasonal terms selected from the stepwise selection in ILI models. The number of excess deaths due to influenza was defined as the difference between predicted number of deaths and baseline number of deaths. The seasonal number of excess deaths due to influenza was summed for each respiratory season. The weekly number of excess hospitalizations and excess mortality due to influenza were estimated as the difference between the expected number and mortality from the full model and baseline hospitalizations and mortality respectively. Then, weekly excess hospitalization and mortality were summed for each July-June respiratory season to obtain excess hospitalization and mortality estimates.

## Supporting Tables

**Table S1: Performance of the models used to estimate excess influenza-associated hospitalization and mortality**

| **Hospitalization, 2010-2018 seasons** | | | | | | | | |
| --- | --- | --- | --- | --- | --- | --- | --- | --- |
| **Age group (years)** | **P&I** | | **Respiratory causes** | | **Cardiac causes** | | **All Causes** | |
|  | **Corr** | **MAPE** | **Corr** | **MAPE** | **Corr** | **MAPE** | **Corr** | **MAPE** |
| **0-4** | 0.97 | 11% | 0.97 | 7% | 0.80 | 7% | 0.92 | 11% |
| **5-19** | 0.93 | 14% | 0.86 | 8% | 0.82 | 4% | 0.89 | 14% |
| **20-49** | 0.96 | 7% | 0.94 | 4% | 0.94 | 5% | 0.91 | 7% |
| **50-64** | 0.98 | 4% | 0.97 | 3% | 0.93 | 4% | 0.90 | 4% |
| **65-74** | 0.97 | 5% | 0.98 | 3% | 0.93 | 4% | 0.91 | 5% |
| **75-84** | 0.98 | 5% | 0.98 | 3% | 0.93 | 3% | 0.91 | 5% |
| **85+** | 0.98 | 5% | 0.98 | 4% | 0.94 | 2% | 0.94 | 5% |
| **Mortality, 2000-2015 seasons** | | | | | | | | |
| **Age group (years)** | **P&I** | | **Respiratory causes** | | **Cardiac causes** | | **All Causes** | |
|  | **Corr** | **MAPE** | **Corr** | **MAPE** | **Corr** | **MAPE** | **Corr** | **MAPE** |
| **0-4** | 0.69 | 46% | 0.76 | 45% | 0.49 | 54% | 0.85 | 6% |
| **5-19** | 0.70 | 53% | 0.56 | 46% | 0.48 | 44% | 0.91 | 8% |
| **20-49** | 0.80 | 35% | 0.80 | 16% | 0.91 | 6% | 0.98 | 2% |
| **50-64** | 0.86 | 18% | 0.89 | 8% | 0.93 | 4% | 0.91 | 2% |
| **65-74** | 0.87 | 14% | 0.92 | 7% | 0.99 | 3% | 0.98 | 2% |
| **75-84** | 0.91 | 9% | 0.94 | 6% | 0.99 | 3% | 0.97 | 2% |
| **85+** | 0.92 | 9% | 0.93 | 7% | 0.98 | 3% | 0.96 | 3% |

Corrr: correlation coefficient; MAPE: Mean Absolute Percentage Error; P&I: Pneumonia & Influenza

**Table S2: Estimated excess influenza-related mortality per cause and age group in France, 2010-1015 seasons**

| **Epidemic Seasons** | **P&I** | | | **Respiratory causes** | | | **Cardiac causes** | | | **All-causes** | | |
| --- | --- | --- | --- | --- | --- | --- | --- | --- | --- | --- | --- | --- |
|  | **N** | **CI_95%_** | **Rate**† | **N** | **CI_95%_** | **Rate**† | **N** | **CI_95%_** | **Rate**† | **N** | **CI_95%_** | **Rate**† |
| **All ages** | | | | | | | | | | | | |
| **2010-11** | 394 | (162-759) | 0.6 | 565 | (239-1,264) | 0.9 | 298 | (78-823) | 0.5 | 3,538 | (1,554-6,684) | 5.6 |
| **2011-12** | 1,714 | (1,348-2,117) | 2.7 | 3,656 | (2,944-4,425) | 5.8 | 3,925 | (2,802-5,085) | 6.2 | 14,414 | (11,291-17,687) | 22.7 |
| **2012-13** | 1,499 | (1,114-19,16) | 2.4 | 2,982 | (2,238-3,770) | 4.7 | 3,929 | (2,780-5,111) | 6.2 | 13,050 | (9,781-16,389) | 20.5 |
| **2013-14** | 49 | (21-311) | 0.1 | 37 | (2-358) | 0.1 | 180 | (6-1,054) | 0.3 | 166 | (9-1,505) | 0.3 |
| **2014-15** | 2,759 | (23,44-3,207) | 4.3 | 4,692 | (3,940-5,489) | 7.3 | 4,898 | (3,851-5,977) | 7.6 | 17,113 | (14,032-20,265) | 26.6 |
| **65+** | | | | | | | | | | | | |
| **2010-11** | 229 | (37-548) | 2.1 | 258 | (0-881) | 2.4 | 138 | (25-538) | 1.3 | 2376 | (803-5103) | 22.3 |
| **2011-12** | 1,690 | (1,345-2,061) | 15.8 | 3,598 | (2,923-4,310) | 33.7 | 3,653 | (2,646-4,686) | 34.2 | 13,344 | (10583-16168) | 125.1 |
| **2012-13** | 1,376 | (1,028-1,749) | 12.9 | 2,729 | (2,052-3,441) | 25.6 | 3,609 | (2,581-4,663) | 33.8 | 11,570 | (8752-14447) | 108.5 |
| **2013-14** | - | - | - | 29 | (0-298) | 0.3 | 81 | (0-833) | 0.8 | 117 | (0-1179) | 1.1 |
| **2014-15** | 2,618 | (2,238-3,024) | 24.5 | 4,453 | (3,765-5,178) | 41.7 | 4,566 | (3,625-5,531) | 42.8 | 16,139 | (13472-18868) | 151.3 |

† Rate per 100 000 persons

CI_95%_: 95% Confidence interval; P&I: Pneumonia & Influenza
