# Supplementary material for: Estimating the burden of influenza‐related and associated hospitalizations and deaths in France: An eight‐season data study, 2010–2018
Source: Influenza Other Respir Viruses. 2022 Jan 10;16(4):717–25. doi: 10.1111/irv.12962 (PMC9178052; doi:10.1111/irv.12962)
Supplement: Supplementary file 2 — Table S1: Performance of the models used to estimate excess influenza‐associated hospitalization and mortality Table S2: Estimated excess influenza‐related mortality per cause and age group in France, 2010–1015 seasons [file IRV-16-717-s001.docx]

Table S1: Performance of the models used to estimate excess influenza-associated hospitalization and mortality

| Hospitalization, 2010-2018 seasons | | | | | | | | |
| --- | --- | --- | --- | --- | --- | --- | --- | --- |
| **Age group (years)** | **P&I** | | **Respiratory causes** | | **Cardiac causes** | | **All Causes** | |
|  | **Corr** | **MAPE** | **Corr** | **MAPE** | **Corr** | **MAPE** | **Corr** | **MAPE** |
| **0-4** | 0.97 | 11% | 0.97 | 7% | 0.80 | 7% | 0.92 | 11% |
| **5-19** | 0.93 | 14% | 0.86 | 8% | 0.82 | 4% | 0.89 | 14% |
| **20-49** | 0.96 | 7% | 0.94 | 4% | 0.94 | 5% | 0.91 | 7% |
| **50-64** | 0.98 | 4% | 0.97 | 3% | 0.93 | 4% | 0.90 | 4% |
| **65-74** | 0.97 | 5% | 0.98 | 3% | 0.93 | 4% | 0.91 | 5% |
| **75-84** | 0.98 | 5% | 0.98 | 3% | 0.93 | 3% | 0.91 | 5% |
| **85+** | 0.98 | 5% | 0.98 | 4% | 0.94 | 2% | 0.94 | 5% |
| **Mortality, 2000-2015 seasons** | | | | | | | | |
| **Age group (years)** | **P&I** | | **Respiratory causes** | | **Cardiac causes** | | **All Causes** | |
|  | **Corr** | **MAPE** | **Corr** | **MAPE** | **Corr** | **MAPE** | **Corr** | **MAPE** |
| **0-4** | 0.69 | 46% | 0.76 | 45% | 0.49 | 54% | 0.85 | 6% |
| **5-19** | 0.70 | 53% | 0.56 | 46% | 0.48 | 44% | 0.91 | 8% |
| **20-49** | 0.80 | 35% | 0.80 | 16% | 0.91 | 6% | 0.98 | 2% |
| **50-64** | 0.86 | 18% | 0.89 | 8% | 0.93 | 4% | 0.91 | 2% |
| **65-74** | 0.87 | 14% | 0.92 | 7% | 0.99 | 3% | 0.98 | 2% |
| **75-84** | 0.91 | 9% | 0.94 | 6% | 0.99 | 3% | 0.97 | 2% |
| **85+** | 0.92 | 9% | 0.93 | 7% | 0.98 | 3% | 0.96 | 3% |

Corrr: correlation coefficient; MAPE: Mean Absolute Percentage Error; P&I: Pneumonia & Influenza

Table S2: Estimated excess influenza-related mortality per cause and age group in France, 2010-1015 seasons

| **Epidemic Seasons** | **P&I** | | | **Respiratory causes** | | | **Cardiac causes** | | | **All-causes** | | |
| --- | --- | --- | --- | --- | --- | --- | --- | --- | --- | --- | --- | --- |
|  | **N** | **CI_95%_** | **Rate**† | **N** | **CI_95%_** | **Rate**† | **N** | **CI_95%_** | **Rate**† | **N** | **CI_95%_** | **Rate**† |
| **All ages** | | | | | | | | | | | | |
| **2010-11** | 394 | (162-759) | 0.6 | 565 | (239-1,264) | 0.9 | 298 | (78-823) | 0.5 | 3,538 | (1,554-6,684) | 5.6 |
| **2011-12** | 1,714 | (1,348-2,117) | 2.7 | 3,656 | (2,944-4,425) | 5.8 | 3,925 | (2,802-5,085) | 6.2 | 14,414 | (11,291-17,687) | 22.7 |
| **2012-13** | 1,499 | (1,114-19,16) | 2.4 | 2,982 | (2,238-3,770) | 4.7 | 3,929 | (2,780-5,111) | 6.2 | 13,050 | (9,781-16,389) | 20.5 |
| **2013-14** | 49 | (21-311) | 0.1 | 37 | (2-358) | 0.1 | 180 | (6-1,054) | 0.3 | 166 | (9-1,505) | 0.3 |
| **2014-15** | 2,759 | (23,44-3,207) | 4.3 | 4,692 | (3,940-5,489) | 7.3 | 4,898 | (3,851-5,977) | 7.6 | 17,113 | (14,032-20,265) | 26.6 |
| **65+** | | | | | | | | | | | | |
| **2010-11** | 229 | (37-548) | 2.1 | 258 | (0-881) | 2.4 | 138 | (25-538) | 1.3 | 2376 | (803-5103) | 22.3 |
| **2011-12** | 1,690 | (1,345-2,061) | 15.8 | 3,598 | (2,923-4,310) | 33.7 | 3,653 | (2,646-4,686) | 34.2 | 13,344 | (10583-16168) | 125.1 |
| **2012-13** | 1,376 | (1,028-1,749) | 12.9 | 2,729 | (2,052-3,441) | 25.6 | 3,609 | (2,581-4,663) | 33.8 | 11,570 | (8752-14447) | 108.5 |
| **2013-14** | - | - | - | 29 | (0-298) | 0.3 | 81 | (0-833) | 0.8 | 117 | (0-1179) | 1.1 |
| **2014-15** | 2,618 | (2,238-3,024) | 24.5 | 4,453 | (3,765-5,178) | 41.7 | 4,566 | (3,625-5,531) | 42.8 | 16,139 | (13472-18868) | 151.3 |

† Rate per 100 000 persons

CI_95%_: 95% Confidence interval; P&I: Pneumonia & Influenza

Legend of supporting Figures

Figure S1: Weekly rates for P&I, respiratory, cardiac, and all-causes hospitalizations per age groups in France, July 2010-June 2018.

Observed hospitalization rates (dashed blue line) and predicted hospitalization rates (blue line) based on a Poisson model integrating seasonal terms, time trends, and influenza-like-illness rates. Baseline morbidity rates predicted by the Poisson model in the absence of influenza activity are indicated by a dark blue area.
