# Supplementary figures and images for: Estimating the burden of influenza‐related and associated hospitalizations and deaths in France: An eight‐season data study, 2010–2018
Source: Influenza Other Respir Viruses. 2022 Jan 10;16(4):717–25. doi: 10.1111/irv.12962 (PMC9178052; doi:10.1111/irv.12962)

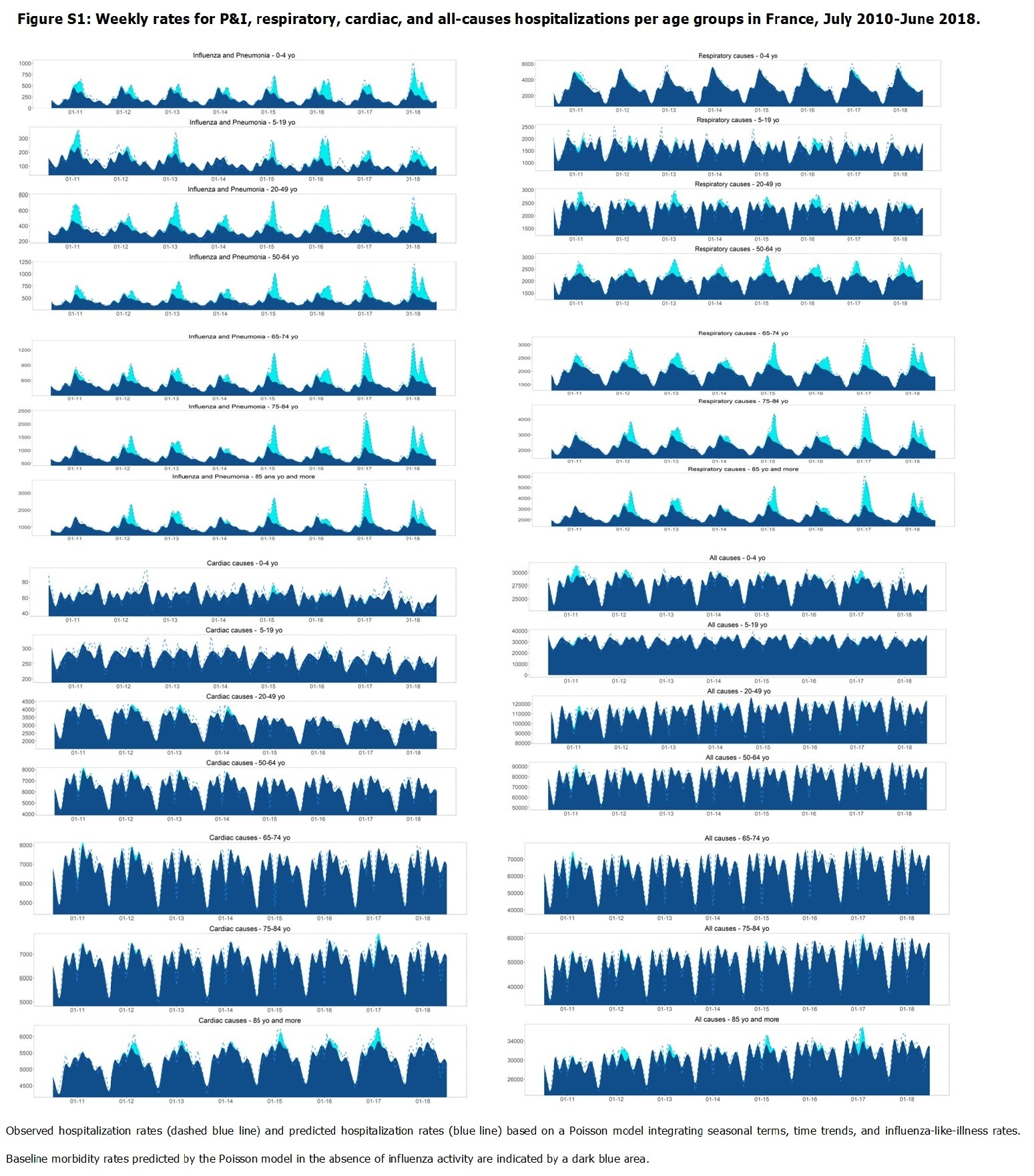

Supplement: Supplementary file 3 — Figure S1: Weekly rates for P&I, respiratory, cardiac, and all‐causes hospitalizations per age groups in France, July 2010–June 2018. Observed hospitalization rates (dashed blue line) and predicted hospitalization rates (blue line) based on a Poisson model integrating seasonal terms, time trends, and influenza‐like‐illness rates. Baseline morbidity rates predicted by the Poisson model in the absence of influenza activity are indicated by a dark blue area. [file IRV-16-717-s003.jpg]
